# The Influence of the Symmetry of Identical Particles on Flight Times

**DOI:** 10.3390/e23121675

**Published:** 2021-12-13

**Authors:** Salvador Miret-Artés, Randall S. Dumont, Tom Rivlin, Eli Pollak

**Affiliations:** 1 Instituto de Física Fundamental, CSIC, Serrano 123, 28006 Madrid, Spain; 2 Department of Chemistry and Chemical Biology, McMaster University, Hamilton, ON L8S 4M1, Canada; dumontr@mcmaster.ca; 3 Chemical and Biological Physics Department, Weizmann Institute of Science, Rehovot 76100, Israel; tom.rivlin@weizmann.ac.il (T.R.); eli.pollak@weizmann.ac.il (E.P.)

**Keywords:** bosons, fermions, flight time distributions, tunneling times

## Abstract

In this work, our purpose is to show how the symmetry of identical particles can influence the time evolution of free particles in the nonrelativistic and relativistic domains as well as in the scattering by a potential δ-barrier. For this goal, we consider a system of either two distinguishable or indistinguishable (bosons and fermions) particles. Two sets of initial conditions have been studied: different initial locations with the same momenta, and the same locations with different momenta. The flight time distribution of particles arriving at a ‘screen’ is calculated in each case from the density and flux. Fermions display broader distributions as compared with either distinguishable particles or bosons, leading to earlier and later arrivals for all the cases analyzed here. The symmetry of the wave function seems to speed up or slow down the propagation of particles. Due to the cross terms, certain initial conditions lead to bimodality in the fermionic case. Within the nonrelativistic domain, and when the short-time survival probability is analyzed, if the cross term becomes important, one finds that the decay of the overlap of fermions is faster than for distinguishable particles which in turn is faster than for bosons. These results are of interest in the short time limit since they imply that the well-known quantum Zeno effect would be stronger for bosons than for fermions. Fermions also arrive earlier and later than bosons when they are scattered by a δ-barrier. Although the particle symmetry does affect the mean tunneling flight time, in the limit of narrow in momentum initial Gaussian wave functions, the mean times are not affected by symmetry but tend to the phase time for distinguishable particles.

## 1. Introduction

It is well understood that the symmetry of indistinguishable particles has a profound influence on their dynamics. A feature which is well documented is the “bunching” of bosons [1,2] and “anti-bunching” of fermions [3,4,5,6,7]. Consider two identical particles, each described for simplicity by an initial Gaussian wavepacket. When the two Gaussians of the two particles are located sufficiently far from each other in phase space, there is no overlap between them and the symmetry of particles plays no role. The fermions and bosons may be considered as two independent distinguishable particles. However, when they come close the symmetry leads to important consequences. Bosons, whose overall function is symmetric with respect to exchange may overlap with each other, hence the interference term ‘increases’ the density between them, causing the “bunching” phenomenon. Fermions on the other hand, due to the anti-symmetry, cannot be located at the same place and the ‘hole’ in the distribution created by the overlap term creates a distancing between the particles, which is understood as the “anti-bunching” effect.

These effects also show up in the temporal dynamics [8,9]. Consider the scattering of two indistinguishable particles on each other and the relative distance (squared) between them as a function of time [8]. As they come closer to each other the distance is reduced and as they move again away it increases. Yet, when comparing such scattering with the exact same potential, incident energy, and so forth. of bosons and fermions, one finds that the distance between the fermions as they separate is larger than that of bosons—another reflection of the bunching and anti-bunching phenomenon [8,9]. Some researchers have even tried to describe the repulsion of fermions in terms of an artificial repulsive potential—the “Pauli potential” [10,11,12,13,14]. In statistical mechanics, this situation leads to the so-called statistical interparticle potential which is temperature-dependent since it is related to what is known as the mean thermal wavelength or thermal de Broglie wavelength [15]. One then speaks about statistical attraction and repulsion for bosons and fermions, respectively. The role of quantum statistics has also been studied in the decay dynamics of a multiple particle state [16].

To the best of our knowledge, the effect of symmetry on flight time distributions [17,18,19,20] for free and scattered particles has not been addressed. The central objective of this present work is to study the interplay between the symmetry of non-interacting particles and their dynamical evolution in time whether as free or scattered particles. A natural consequence of time evolution is the broadening of wavepackets. We find that this broadening serves to further accentuate the bunching and anti-bunching effect found in the initial density. As a result, when one of the two identical particles arrives at a suitably placed screen, its distribution of arrival times will be substantially broadened if it is a fermion as compared to a boson. This implies that a fraction of the fermions would arrive at the screen earlier than bosons and a fraction later. This dynamical magnification of the bunching and anti-bunching effect is absent when considering relativistic identical particles, since in the relativistic regime, wavepackets are hardly broadened in time, so that only the bunching and anti-bunching of the initial density affects the arrival time at the screen. This also implies that one cannot speak of a superluminal effect of early arrivals of fermions, since it is just a mirror, of the corresponding initial spatial distributions.

For the free particle dynamics, we identify a dynamical symmetry (DS) factor which describes how time evolution is affected by the symmetry property of the identical particles. This factor displays opposite phases when considering bosons and fermions. A related question has to do with the survival probability of the initial wavefunction. We shall show that the bosonic survival probability of free particles initially decays slower than that of distinguishable particles while for fermions it decays more rapidly. These results imply that the well-known quantum Zeno effect [21,22,23] would be stronger for bosons than for fermions. Finally, when considering scattering through a delta potential, the particle symmetry does not affect the mean tunneling flight time, which is given by the phase time for the distinguishable particle [19,24].

The paper is organized as follows. In Section 2 we consider the case of free particle propagation in the non-relativistic and relativistic domains. In Section 3 the scattering of identical particles from a delta barrier potential is analyzed in detail since closed analytical expressions can be obtained. Section 4 presents numerical results for free and tunneling dynamics. We consider further generalizations and implications of these results to realistic systems such as neutrons, neutrinos and electrons in Section 5.

## 2. Free Dynamics of Nonrelativistic and Relativistic Identical Particles

### 2.1. General Considerations

We consider two (one dimensional) non-interacting identical particles (with coordinates $x_{1}$ and $x_{2})$ and mass M which may scatter independently from a potential $V\left(x_{j}\right)$. A screen is placed to the right or left of the potential and the arrival time is measured whenever a particle hits the screen. The questions we seek to answer are what is the distribution of times at which one of the particles hits the screen, and what is the mean time it takes to do so, assuming that the mean time exists. The Hamiltonian for a single particle (operators are denoted with carets) is:

(1) $${\hat{H}}_{j}=\frac{{\hat{p}}_{j}^{2}}{2M}+V\left({\hat{x}}_{j}\right),j=1,2,$$

with ${\hat{p}}_{j}$ and ${\hat{x}}_{j}$ the momentum and position operators of the j-th particle respectively. The full Hamiltonian is the sum of the two:

(2) $$\hat{H}={\hat{H}}_{1}+{\hat{H}}_{2}.$$

Initially, the single particle wavefunction will be a coherent state localized about the mean position $x_{ji}$ and mean momentum $p_{ji}$ with width parameter Γ,

(3) $$\Psi_{j}\left(x_{j}\right)={\left(\frac{\Gamma }{\pi }\right)}^{1/4}\exp\left[-\frac{\Gamma {\left(x_{j}-x_{ji}\right)}^{2}}{2}+\frac{i}{\hbar }p_{ji}\left(x_{j}-x_{ji}\right)\right],j=1,2.$$

To simplify, we introduce at this point reduced positions, momenta, and time as:

(4) $$X_{j}=\sqrt{\Gamma }x_{j},K_{j}=\frac{p_{j}}{\hbar \sqrt{\Gamma }},\tau =\frac{\hbar \Gamma }{M}t,$$

so that the single particle initial wavefunction has the form

(5) $$\Psi_{j}\left(X_{j}\right)={\left(\frac{1}{\pi }\right)}^{1/4}\exp\left[-\frac{1}{2}{\left(X_{j}-X_{ji}\right)}^{2}+iK_{ji}\left(X_{j}-X_{ji}\right)\right],j=1,2.$$

The composite wavefunction of the two particles is:

(6) $$\Psi_{\alpha }\left(X_{1},X_{2}\right)=\frac{1}{N_{\alpha }}\left[\Psi_{1}\left(X_{1}\right)\Psi_{2}\left(X_{2}\right)+\cos\left(\pi \alpha \right)\Psi_{2}\left(X_{1}\right)\Psi_{1}\left(X_{2}\right)\right],$$

where the coefficient α is:

(7) $$\alpha =\left(\begin{array}{c} 0\\ 1\\ \frac{1}{2} \end{array}\right)\;\mathrm{for}\;\left(\begin{array}{c} \mathrm{bosons}\\ \mathrm{fermions}\\ \mathrm{distinguishable}\;\mathrm{particles} \end{array}\right).$$

In principle, α can take any other value and then one speaks of anyons.

The corresponding normalization constant is

(8) $$N_{\alpha }^{2}=\left[1+\cos^{2}\left(\pi \alpha \right)+2\cos\left(\pi \alpha \right)\exp\left(-\frac{\Delta_{X}^{2}+\Delta_{K}^{2}}{2}\right)\right],$$

where we used the notation:

(9) $$\Delta_{X}=X_{i2}-X_{i1},\Delta_{K}=K_{i2}-K_{i1},$$

for the differences between the initial mean locations and momenta of the Gaussian wavepackets.

The time-evolved wavefunction is:

(10) $$\begin{array}{ccc} N_{\alpha }\Psi_{\alpha }\left(X_{1},X_{2},\tau \right) & = & \left[\exp\left(-i{\hat{H}}_{1}\tau \right)\Psi_{1}\left(X_{1}\right)\right]\left[\exp\left(-i{\hat{H}}_{2}\tau \right)\Psi_{2}\left(X_{2}\right)\right]\\ & & +\cos\left(\pi \alpha \right)\left[\exp\left(-i{\hat{H}}_{1}\tau \right)\Psi_{2}\left(X_{1}\right)\right]\left[\exp\left(-i{\hat{H}}_{2}\tau \right)\Psi_{1}\left(X_{2}\right)\right]\\ & \equiv & \left[\Psi_{1}\left(X_{1},\tau \right)\Psi_{2}\left(X_{2},\tau \right)+\cos\left(\pi \alpha \right)\Psi_{2}\left(X_{1},\tau \right)\Psi_{1}\left(X_{2},\tau \right)\right]. \end{array}$$

We then put a ‘screen’ at the point $X=X_{f}$ and measure the time at which one of the particles hits the screen. This implies that the density for either one of the particles hitting the screen located at *X* at the time τ is:

(11) $$\rho_{\alpha }\left(X;\tau \right)\equiv \int_{-\infty }^{\infty }dX_{1}\int_{-\infty }^{\infty }dX_{2}{\left|\Psi_{\alpha }\left(X_{1},X_{2},\tau \right)\right|}^{2}\left[\delta \left(X-X_{1}\right)+\delta \left(X-X_{2}\right)\right].$$

Note that $\int_{-\infty }^{\infty }dX\rho_{\alpha }\left(X;\tau \right)=2$ and this is as it should be since we have two particles. This density expresses the fact that first one of the particles hits the screen at X (first delta function) and the second particle is anywhere. Then, another particle hits the screen at X (second delta function).

One typically considers two different measures for the time duration of such an event. One is the density. Alternatively, one may measure the flux of particles hitting the screen and then the time distribution for the particle to reach the screen will be determined by the flux density $J_{\alpha }\left(X;\tau \right)$ defined as:

(12) $$\begin{array}{ccc} J_{\alpha }\left(X;\tau \right) & = & \int_{-\infty }^{\infty }dX_{2}Im\left[\Psi_{\alpha }^{*}\left(X,X_{2},\tau \right)\left(\frac{\partial }{\partial X}\Psi_{\alpha }\left(X,X_{2},\tau \right)\right)\right]\\ & & +\int_{-\infty }^{\infty }dX_{1}Im\left[\Psi_{\alpha }^{*}\left(X_{1},X,\tau \right)\left(\frac{\partial }{\partial X}\Psi_{\alpha }\left(X_{1},X,\tau \right)\right)\right]. \end{array}$$

As also discussed below and in Ref. [19], under suitable conditions the differences between the density and flux transition time distributions disappear when considering a stationary phase approximation and in practice are negligible except when considering their behavior at very long times. The density of a single free particle decays as $\tau^{-1}$ so that the density for a particle at the screen location *X* at time τ decays at long time also as $\tau^{-1}$ (see also below) while the flux density decays at long time as $\tau^{-2}$. Strictly speaking, this implies that the density time distribution of a free particle cannot be normalized to unity and so is not a probability distribution. However, the dependence of the density on the time is typically a bell shaped function which decays almost to 0 by some time $\tau_{f}$ so that we may define a time density probability distribution as:

(13) $$P_{\alpha ,D}\left(\tau ;X,\tau_{f}\right)=\frac{\rho_{\alpha }\left(X;\tau \right)}{\int_{0}^{\tau_{f}}d\tau \rho_{\alpha }\left(X;\tau \right)}\equiv N_{\alpha ,D}\left(\tau_{f}\right)\rho_{\alpha }\left(X_{f};\tau \right).$$

The time integral of the flux density distribution converges (since it goes as $\tau^{-2}$ at long times) so that the flux density transition path time distribution at the screen located at *X* is defined as:

(14) $$P_{\alpha ,J}\left(\tau ;X\right)=\frac{J_{\alpha }\left(X;\tau \right)}{\int_{0}^{\infty }d\tau J_{\alpha }\left(X;\tau \right)}.$$

We note that these definitions are not the same as the so-called presence time distributions, since the time interval we consider is $\left[0,\infty \right]$ while the presence times are defined for the time interval $\left[-\infty ,\infty \right]$. The differences are not technical, as discussed in Ref. [19].

When a particle is scattered on a potential, the density decays at long times at least as $\tau^{-3}$ [25,26], so that there is no difficulty in defining the density probability distribution by allowing $\tau_{f}\to \infty$ and in addition the mean transition path time,

(15) $${\langle \tau \rangle }_{\alpha ,A}=\int_{0}^{\infty }d\tau \tau P_{\alpha ,A}\left(\tau ;X\right),A=D,J,$$

is also finite. In Section IV, we provide an example of a numerical comparison of the density and flux-density distributions for a freely evolving particle which shows that the differences between them are small. In a recent publication, we have also shown [19] that the information given by the density and the flux time distributions for a single particle is almost the same so that, in practice, one may resort to the simpler density time distribution and the same conclusions would hold for the flux-density distributions.

### 2.2. Symmetry and Free Particles

The single free particle (V=0) time evolved wavefunction is:

(16) $$\Psi_{j}\left(X,X_{ji},K_{ji},\tau \right)=\frac{1}{\sqrt{\left(1+i\tau \right)}}{\left(\frac{1}{\pi }\right)}^{1/4}\exp\left(-\frac{1}{2}\frac{{\left[\left(X-X_{ji}\right)-iK_{ji}\right]}^{2}}{\left(1+i\tau \right)}-\frac{K_{ji}^{2}}{2}\right),j=1,2,$$

and the density is:

(17) $${\left|\Psi_{j}\left(X,X_{ji},K_{ji},\tau \right)\right|}^{2}=\frac{1}{\sqrt{\pi \left(1+\tau^{2}\right)}}\exp\left(-\frac{{\left(X-X_{ji}-K_{ji}\tau \right)}^{2}}{\left(1+\tau^{2}\right)}\right),j=1,2.$$

The single free particle time-dependent density has a maximum at the (free particle) time $\tau_{\max}=\left(X-X_{ji}\right)/K_{ji}$. It is normalized when integrating over the position *X* but diverges when integrating over the time due to the long time tail, which goes as 1/τ.

After some Gaussian integrations, using the shorthand notation,

(18) $$X_{i}\equiv \frac{X_{i1}+X_{i2}}{2},K_{i}\equiv \frac{K_{i1}+K_{i2}}{2}$$

and

(19) $$X\left(\tau \right)=X-X_{i}-K_{i}\tau ,\Delta \left(\tau \right)=\Delta_{X}+\tau \Delta_{K},$$

one finds that the general expression for the one particle density (see Equation (11)) at the screen location *X* is:

(20) $$\begin{array}{ccc} & & \rho_{\alpha }\left(X;\tau \right)\\ & = & \frac{2}{N_{\alpha }^{2}\sqrt{\pi \left(1+\tau^{2}\right)}}\exp\left(-\frac{X{\left(\tau \right)}^{2}}{\left(1+\tau^{2}\right)}-\frac{\Delta {\left(\tau \right)}^{2}}{4\left(1+\tau^{2}\right)}\right)\\ & & \left[\cosh\left(\frac{X\left(\tau \right)\Delta \left(\tau \right)}{\left(1+\tau^{2}\right)}\right)\left[1+\cos^{2}\left(\pi \alpha \right)\right]+2\cos\left(\pi \alpha \right)e^{-\frac{\Delta_{X}^{2}+\Delta_{K}^{2}}{4}}\cos\left(\frac{\left(\Delta_{K}-\tau \Delta_{X}\right)X\left(\tau \right)}{\left(1+\tau^{2}\right)}\right)\right]. \end{array}$$

For the flux (see Equation (12)), the expression is a bit more involved, one finds:

(21) $$\begin{array}{ccc} & & J_{\alpha }\left(X;\tau \right)\\ & = & \frac{\left[K_{i}+\tau \left(X-X_{i}\right)\right]}{\left(1+\tau^{2}\right)}\rho_{\alpha }\left(X,\tau \right)\\ & & +\frac{\left(\Delta_{K}-\tau \Delta_{X}\right)\left[1+\cos^{2}\left(\pi \alpha \right)\right]}{\sqrt{\pi }N_{\alpha }^{2}{\left(1+\tau^{2}\right)}^{3/2}}\exp\left(-\frac{\left(X^{2}\left(\tau \right)+\frac{\Delta^{2}\left(\tau \right)}{4}\right)}{\left(1+\tau^{2}\right)}\right)\sinh\left(\frac{X\left(\tau \right)\Delta \left(\tau \right)}{\left(1+\tau^{2}\right)}\right)\\ & & +\frac{2\cos\left(\pi \alpha \right)\Delta \left(\tau \right)}{\sqrt{\pi }N_{\alpha }^{2}{\left(1+\tau^{2}\right)}^{3/2}}\exp\left(-\frac{\left(X^{2}\left(\tau \right)+\frac{\Delta^{2}\left(\tau \right)}{4}\right)}{\left(1+\tau^{2}\right)}-\frac{\Delta_{X}^{2}+\Delta_{K}^{2}}{4}\right)\sin\left(\frac{X\left(\tau \right)\left(\Delta_{K}-\tau \Delta_{X}\right)}{\left(1+\tau^{2}\right)}\right). \end{array}$$

The long time limit for the density is:

(22) $$\begin{array}{ccc} & & \lim_{\tau \to \infty }\rho_{\alpha }\left(X;\tau \right)\\ & = & \frac{2}{\tau N_{\alpha }^{2}\sqrt{\pi }}\exp\left(-K_{i}^{2}-\frac{\Delta_{K}^{2}}{4}\right)\\ & & \cdot \left[\cosh\left(K_{i}\Delta_{K}\right)\left[1+\cos^{2}\left(\pi \alpha \right)\right]+2\cos\left(\pi \alpha \right)e^{-\frac{\Delta_{X}^{2}+\Delta_{K}^{2}}{4}}\cos\left(\Delta_{X}K_{i}\right)\right] \end{array}$$

and for the flux it is:

(23) $$\begin{array}{ccc} & & \lim_{\tau \to \infty }J_{\alpha }\left(X;\tau \right)\\ & = & \frac{1}{\tau^{2}}\frac{\left[1+\cos^{2}\left(\pi \alpha \right)\right]}{N_{\alpha }^{2}\sqrt{\pi }}\exp\left(-K_{i}^{2}-\frac{\Delta_{K}^{2}}{4}\right)\left[2\left(X-X_{i}\right)\cosh\left(K_{i}\Delta_{K}\right)+\Delta_{X}\sinh\left(K_{i}\Delta_{K}\right)\right]\\ & & +\frac{1}{\tau^{2}}\frac{2\cos\left(\pi \alpha \right)}{N_{\alpha }^{2}\sqrt{\pi }}\exp\left(-K_{i}^{2}-\frac{\Delta_{K}^{2}}{4}-\frac{\Delta_{X}^{2}+\Delta_{K}^{2}}{4}\right)\left[2\left(X-X_{i}\right)\cos\left(\Delta_{X}K_{i}\right)+\Delta_{K}\sin\left(K_{i}\Delta_{X}\right)\right], \end{array}$$

demonstrating explicitly the $\tau^{-1}$ and $\tau^{-2}$ asymptotic dependence of the density and flux, and that the magnitude of the tails may be extremely small if the mean (reduced) momentum $K_{i}$ is somewhat larger than unity.

#### 2.2.1. Distinguishable Particles

In this case the symmetry parameter is α=1/2 and one finds the relatively simple and expected result:

(24) $$\begin{array}{ccc} & & \rho_{1/2}\left(X;\tau \right)\\ & = & \frac{1}{\sqrt{\pi \left(1+\tau^{2}\right)}}\left(\exp\left[-\frac{{\left(X\left(\tau \right)+\frac{\Delta \left(\tau \right)}{2}\right)}^{2}}{\left(1+\tau^{2}\right)}\right]+\exp\left[-\frac{{\left(X\left(\tau \right)-\frac{\Delta \left(\tau \right)}{2}\right)}^{2}}{\left(1+\tau^{2}\right)}\right]\right). \end{array}$$

The density of the first Gaussian corresponding to particle 1 maximizes at $\tau =\left(X-X_{1i}\right)/K_{1i}$ while the second Gaussian, corresponding to the second particle will maximize at $\tau =\left(X-X_{2i}\right)/K_{2i}$ as expected for two independent but distinguishable particles. For the flux one finds:

(25) $$\begin{array}{ccc} & & J_{1/2}\left(X;\tau \right)\\ & = & \frac{1}{{\left(1+\tau^{2}\right)}^{3/2}\sqrt{\pi }}\exp\left(-\frac{\left(X^{2}\left(\tau \right)+\frac{\Delta^{2}\left(\tau \right)}{4}\right)}{\left(1+\tau^{2}\right)}\right)\\ & & \left[2\left[K_{i}+\tau \left(X-X_{i}\right)\right]\cosh\left(\frac{X\left(\tau \right)\Delta \left(\tau \right)}{\left(1+\tau^{2}\right)}\right)+\left(\Delta_{K}-\tau \Delta_{x}\right)\sinh\left(\frac{X\left(\tau \right)\Delta \left(\tau \right)}{\left(1+\tau^{2}\right)}\right)\right]. \end{array}$$

The single particle density may be used to define a DS density factor, which describes how the time evolution of the density is affected through the symmetry of the particles:

(26) $$F_{DS,\alpha }\left(X;\tau \right)\equiv \frac{\rho_{\alpha }\left(X;\tau \right)}{\rho_{1/2}\left(X;\tau \right)}.$$

#### 2.2.2. Bosons

The boson single particle density (α=0) is:

(27) $$\begin{array}{ccc} & & \rho_{0}\left(X;\tau \right)\\ & = & \frac{2}{\left[1+\exp\left(-\frac{\Delta_{X}^{2}+\Delta_{K}^{2}}{2}\right)\right]\sqrt{\pi \left(1+\tau^{2}\right)}}\exp\left(-\frac{X{\left(\tau \right)}^{2}}{\left(1+\tau^{2}\right)}-\frac{\Delta {\left(\tau \right)}^{2}}{4\left(1+\tau^{2}\right)}\right)\\ & & \left[\cosh\left(\frac{X\left(\tau \right)\Delta \left(\tau \right)}{\left(1+\tau^{2}\right)}\right)+e^{-\frac{\Delta_{X}^{2}+\Delta_{K}^{2}}{4}}\cos\left(\frac{\left(\Delta_{K}-\tau \Delta_{X}\right)X\left(\tau \right)}{\left(1+\tau^{2}\right)}\right)\right]. \end{array}$$

The flux density at the screen *X* is:

(28) $$\begin{array}{ccc} & & J_{0}\left(X;\tau \right)\\ & = & \frac{\left[K_{i}+\tau \left(X-X_{i}\right)\right]}{\left(1+\tau^{2}\right)}\rho_{0}\left(X,\tau \right)\\ & & +\frac{2}{\sqrt{\pi }N_{\alpha }^{2}{\left(1+\tau^{2}\right)}^{3/2}}\exp\left(-\frac{\left(X^{2}\left(\tau \right)+\frac{\Delta^{2}\left(\tau \right)}{4}\right)}{\left(1+\tau^{2}\right)}\right)\\ & & \cdot \left[\left(\Delta_{K}-\tau \Delta_{X}\right)\sinh\left(\frac{X\left(\tau \right)\Delta \left(\tau \right)}{\left(1+\tau^{2}\right)}\right)+\exp\left(-\frac{\Delta_{X}^{2}+\Delta_{K}^{2}}{4}\right)\Delta \left(\tau \right)\sin\left(\frac{X\left(\tau \right)\left(\Delta_{K}-\tau \Delta_{X}\right)}{\left(1+\tau^{2}\right)}\right)\right] \end{array}$$

and the DS factor for the density is:

(29) $$\begin{array}{ccc} & & F_{DS,0}\left(X;\tau \right)=\frac{1}{\left[1+\exp\left(-\frac{\Delta_{X}^{2}+\Delta_{K}^{2}}{2}\right)\right]}\\ & & \cdot \left[1+{\left[\cosh\left(\frac{X\left(\tau \right)\Delta \left(\tau \right)}{\left(1+\tau^{2}\right)}\right)\right]}^{-1}\exp\left(-\frac{\Delta_{X}^{2}+\Delta_{K}^{2}}{4}\right)\cos\left(\frac{\left(\Delta_{K}-\tau \Delta_{X}\right)X\left(\tau \right)}{\left(1+\tau^{2}\right)}\right)\right]. \end{array}$$

If the two bosons are initially placed such that their Gaussian centers are identical ($\Delta_{X}=\Delta_{K}=0$), the time dependent density is the same as for the single distinguishable particle and the dynamical symmetry factor reduces to unity. Similarly, if the initial distance between the two wavepackets is sufficiently large, the interference cross term will vanish and the result will reduce to the distinguishable particle case.

#### 2.2.3. Fermions

The fermionic density (α=1) is:

(30) $$\begin{array}{ccc} & & \rho_{1}\left(X;\tau \right)\\ & = & \frac{2}{\left[1-\exp\left(-\frac{\Delta_{X}^{2}+\Delta_{K}^{2}}{2}\right)\right]\sqrt{\pi \left(1+\tau^{2}\right)}}\exp\left(-\frac{X{\left(\tau \right)}^{2}}{\left(1+\tau^{2}\right)}-\frac{\Delta {\left(\tau \right)}^{2}}{4\left(1+\tau^{2}\right)}\right)\\ & & \left[\cosh\left(\frac{X\left(\tau \right)\Delta \left(\tau \right)}{\left(1+\tau^{2}\right)}\right)-e^{-\frac{\Delta_{X}^{2}+\Delta_{K}^{2}}{4}}\cos\left(\frac{\left(\Delta_{K}-\tau \Delta_{X}\right)X\left(\tau \right)}{\left(1+\tau^{2}\right)}\right)\right] \end{array}$$

and the flux-density is:

(31) $$\begin{array}{ccc} & & J_{1}\left(X;\tau \right)\\ & = & \frac{\left[K_{i}+\tau \left(X-X_{i}\right)\right]}{\left(1+\tau^{2}\right)}\rho_{1}\left(X,\tau \right)\\ & & +\frac{1}{\sqrt{\pi }\left[1-\exp\left(-\frac{\Delta_{X}^{2}+\Delta_{K}^{2}}{2}\right)\right]{\left(1+\tau^{2}\right)}^{3/2}}\exp\left(-\frac{\left(X^{2}\left(\tau \right)+\frac{\Delta^{2}\left(\tau \right)}{4}\right)}{\left(1+\tau^{2}\right)}\right)\\ & & \cdot \left[\left(\Delta_{K}-\tau \Delta_{X}\right)\sinh\left(\frac{X\left(\tau \right)\Delta \left(\tau \right)}{\left(1+\tau^{2}\right)}\right)+\exp\left(-\frac{\Delta_{X}^{2}+\Delta_{K}^{2}}{4}\right)\Delta \left(\tau \right)\sin\left(\frac{X\left(\tau \right)\left(\Delta_{K}-\tau \Delta_{X}\right)}{\left(1+\tau^{2}\right)}\right)\right]. \end{array}$$

In the limit that $\Delta_{X}=\Delta_{K}=0$ both the numerators and denominators vanish but their ratio does not. For example,

(32) $$\begin{array}{ccc} & & \lim_{\Delta_{X},\Delta_{K}\to 0}\rho_{1}\left(X;\tau \right)\\ & = & \frac{1}{\sqrt{\pi \left(1+\tau^{2}\right)}}\exp\left(-\frac{X{\left(\tau \right)}^{2}}{\left(1+\tau^{2}\right)}\right)\left[1+\frac{2X^{2}\left(\tau \right)}{\left(1+\tau^{2}\right)}\right]. \end{array}$$

More specifically, at time τ=0, we have with $K_{1i}=K_{2i}=K_{i}$ and $\Delta_{X}=X_{2i}-X_{1i}$:

(33) $$\begin{array}{ccc} & & \lim_{\Delta_{X}\to 0}\Psi \left(X_{1},X_{2},0\right)=-\frac{1}{\sqrt{\pi }}\left(X_{2}-X_{1}\right)\\ & & \cdot \exp\left[iK_{i}\left(X_{1}-X_{1i}\right)+iK_{i}\left(X_{2}-X_{1i}\right)-\frac{1}{2}\left[{\left(X_{1}-X_{1i}\right)}^{2}+{\left(X_{2}-X_{1i}\right)}^{2}\right]\right], \end{array}$$

and this vanishes if $X_{1}=X_{2}$. Fermions cannot exist at the same point in phase space. Note, however, that:

(34) $$\int_{-\infty }^{\infty }dX_{1}\int_{-\infty }^{\infty }dX_{2}\lim_{\Delta_{i}\to 0}{\left|\Psi \left(X_{1},X_{2},0\right)\right|}^{2}=1,$$

as it should be. There is no difficulty in preparing an initial wavefunction in the fermionic case even if both wavepackets are localized around the same centers both in coordinate and momentum space. The density vanishes at one point only.

The DS factor for the fermionic density is given by:

(35) $$\begin{array}{ccc} F_{DS,1}\left(X;\tau \right) & = & \frac{1}{\left[1-\exp\left(-\frac{\Delta_{X}^{2}+\Delta_{K}^{2}}{2}\right)\right]}\\ & & \cdot \left[1-e^{-\frac{\Delta_{X}^{2}+\Delta_{K}^{2}}{4}}{\left[\cosh\left(\frac{X\left(\tau \right)\Delta \left(\tau \right)}{\left(1+\tau^{2}\right)}\right)\right]}^{-1}\cos\left(\frac{\left(\Delta_{K}-\tau \Delta_{X}\right)X\left(\tau \right)}{\left(1+\tau^{2}\right)}\right)\right]. \end{array}$$

### 2.3. Survival Probability and Symmetry

Denoting the overlap of different single particle wavefunctions as:

(36) $$S_{ij}\left(\tau \right)=\int_{-\infty }^{\infty }dX\Psi_{i}^{*}\left(X\right)\Psi_{j}\left(X,\tau \right),\;\;i,j=1,2,$$

we find that for the free particle evolution,

(37) $$S_{jj}\left(\tau \right)=\sqrt{\frac{2}{\left(2+i\tau \right)}}\exp\left(-\frac{i\tau K_{ji}^{2}}{\left(2+i\tau \right)}\right),\;\;j=1,2,$$

so that the time dependent overlap squared, termed also the survival probability is of Gaussian form:

(38) $${\left|S_{j}\left(\tau \right)\right|}^{2}=\sqrt{\frac{4}{\left(4+\tau^{2}\right)}}\exp\left(-\frac{2\tau^{2}K_{ji}^{2}}{\left(4+\tau^{2}\right)}\right),\;\;i,j=1,2,$$

while

(39) $$S_{12}\left(\tau \right)=\frac{1}{\sqrt{\left(1+i\tau /2\right)}}\exp\left(-\frac{\Delta_{K}^{2}}{4}-K_{i}^{2}-\frac{1}{2}\frac{{\left[\Delta_{X}+2iK_{i}\right]}^{2}}{\left(2+i\tau \right)}\right)$$

and

(40) $$S_{21}\left(\tau \right)=\frac{1}{\sqrt{\left(1+i\tau /2\right)}}\exp\left(-\frac{\Delta_{K}^{2}}{4}-K_{i}^{2}\right)\exp\left(-\frac{1}{2}\frac{{\left[\Delta_{X}-2iK_{i}\right]}^{2}}{\left(2+i\tau \right)}\right).$$

The overlap of the two particle initial wavefunction with its time evolved form is then readily seen to be:

(41) $$S_{\alpha }\left(\tau \right)=\frac{1}{N_{\alpha }^{2}}\left(\left[S_{11}\left(\tau \right)S_{22}\left(\tau \right)\right]\left[1+\cos^{2}\left(\pi \alpha \right)\right]+2\cos\left(\pi \alpha \right)S_{21}\left(\tau \right)S_{12}\left(\tau \right)\right),$$

so that with some algebra one finds that the survival probability for identical particles is:

(42) $${\left|S_{\alpha }\left(\tau \right)\right|}^{2}={\left|S_{1/2}\left(\tau \right)\right|}^{2}F_{\alpha }\left(\tau \right),$$

with the survival symmetry factor $F_{\alpha }\left(\tau \right)$ given by:

(43) $$\begin{array}{ccc} F_{\alpha }\left(\tau \right) & = & \frac{1}{N_{\alpha }^{4}}{\left[1+\cos^{2}\left(\pi \alpha \right)\right]}^{2}+4\cos^{2}\left(\pi \alpha \right)\exp\left(-\frac{4\left(\Delta_{K}^{2}+\Delta_{X}^{2}\right)}{\left(4+\tau^{2}\right)}\right)\\ & + & \frac{4\cos\left(\pi \alpha \right)\left[1+\cos^{2}\left(\pi \alpha \right)\right]}{N_{\alpha }^{4}}\exp\left(-\frac{2\left(\Delta_{K}^{2}+\Delta_{X}^{2}\right)}{\left(4+\tau^{2}\right)}\right)\cos\left[\frac{\tau \left(\Delta_{K}^{2}+\Delta_{X}^{2}\right)}{\left(4+\tau^{2}\right)}\right], \end{array}$$

and the distinguishable particle survival probability as:

(44) $${\left|S_{1/2}\left(\tau \right)\right|}^{2}=\frac{4}{\left(4+\tau^{2}\right)}\exp\left[-\frac{4\tau^{2}}{\left(4+\tau^{2}\right)}\left(K_{i}^{2}+\frac{\Delta_{K}^{2}}{4}\right)\right].$$

It is then of interest to study the survival probability in some limits. First, we note that when the initial distances between the wavepackets are sufficiently large, such that $\Delta_{X}^{2}+\Delta_{K}^{2}\gg 1$, then for times shorter than $∼\sqrt{\Delta_{X}^{2}+\Delta_{K}^{2}}$, this overlap function reduces to unity. This is what is expected: when the initial distance between the particles is large, they behave as independent distinguishable particles. The interesting case is when the initial distances between the two wavepackets are small and the interference term is no longer negligible at short times. One finds:

(45) $$\begin{array}{ccc} \lim_{\Delta_{K}^{2}+\Delta_{X}^{2}\to 0}\frac{{\left|S_{0}\left(\tau \right)\right|}^{2}}{{\left|S_{1/2}\left(\tau \right)\right|}^{2}} & = & 1+O\left(\Delta_{K}^{2}+\Delta_{X}^{2}\right) \end{array}$$

(46) $$\begin{array}{ccc} \lim_{\Delta_{K}^{2}+\Delta_{X}^{2}\to 0}\frac{{\left|S_{1}\left(\tau \right)\right|}^{2}}{{\left|S_{1/2}\left(\tau \right)\right|}^{2}} & = & \frac{4}{\left(4+\tau^{2}\right)}+O\left(\Delta_{K}^{2}+\Delta_{X}^{2}\right), \end{array}$$

implying that the survival probability for fermions decays more rapidly than that of bosons. In other words, when the distance in phase space between the centers of the two particles is small, which is the case when the interference term becomes most important, one finds that the decay of the overlap of fermions is faster than distinguishable particles which in turn is faster than bosons.

In the short time limit, one has for bosons that:

(47) $$\begin{array}{ccc} & & \lim_{\tau \to 0}\frac{{\left|S_{0}\left(\tau \right)\right|}^{2}}{{\left|S_{1/2}\left(\tau \right)\right|}^{2}}\\ & = & 1+\frac{\left(\Delta_{K}^{2}+\Delta_{X}^{2}\right)\tau^{2}}{N_{0}^{4}}\exp\left(-\frac{\left(\Delta_{K}^{2}+\Delta_{X}^{2}\right)}{2}\right)\left(1-\frac{\left(\Delta_{K}^{2}+\Delta_{X}^{2}\right)}{4}\right)+\exp\left(-\frac{\left(\Delta_{K}^{2}+\Delta_{X}^{2}\right)}{2}\right), \end{array}$$

while for fermions

(48) $$\begin{array}{ccc} & & \lim_{\tau \to 0}\frac{{\left|S_{1}\left(\tau \right)\right|}^{2}}{{\left|S_{1/2}\left(\tau \right)\right|}^{2}}\\ & = & 1-\frac{\left(\Delta_{K}^{2}+\Delta_{X}^{2}\right)\tau^{2}}{N_{1}^{4}}\exp\left(-\frac{\left(\Delta_{K}^{2}+\Delta_{X}^{2}\right)}{2}\right)\left[\left(1-\frac{\left(\Delta_{K}^{2}+\Delta_{X}^{2}\right)}{4}\right)-\exp\left(-\frac{\left(\Delta_{K}^{2}+\Delta_{X}^{2}\right)}{2}\right)\right], \end{array}$$

demonstrating that, as long as $\left(\Delta_{K}^{2}+\Delta_{X}^{2}\right)<4$, the quantum Zeno effect [21,22,23] would be stronger for bosons than for fermions, as the fermionic survival probability decays faster at short times.

### 2.4. Free Dynamics of Relativistic Identical Particles

To investigate the relativistic regime, we consider relativistic electrons and photons. The wavepackets describing the bosons—the photons—travel dispersion-free at the speed of light. The wavepackets describing the fermions—the electrons—are four component spinors with time evolution determined by the Dirac equation. As we consider only the free particle motion of two non-interacting (except via particle statistics) electrons, spin is conserved and the wavepackets reduce to two component spinors. Relativistic wavepacket propagation is much like non-relativistic propagation except that the velocity is no longer directly proportional to wavenumber–the former asymptotes to the speed of light—and wavepacket broadening is greatly suppressed due to the dispersion relation—quadratic in the non-relativistic case—approaching linearity. In particular, the time scale for wavepacket broadening scales with $\gamma^{2}$, where $\gamma =1/\sqrt{1-v^{2}/c^{2}}$. (See Eq. (2.19) in [24].) For example, if v=0.99c, broadening takes 50 times longer than it does for non-relativistic velocities.

The single free relativistic electron time evolved wavefunction, in the highly accurate (for cases we considered) steepest descent approximation, is:

(49) $$\Psi_{j}\left(X,X_{i},K_{i},\tau \right)=\frac{\hat{u}}{\sqrt{\left(1+i\left(\tau /\gamma^{2}\right)\right)}}{\left(\frac{1}{\pi }\right)}^{1/4}\exp\left(-\frac{1}{2}\frac{{\left[\left(X_{j}-X_{ji}\right)-iK_{ji}\right]}^{2}}{\left(1+i\left(\tau /\gamma^{2}\right)\right)}-\frac{K_{ji}^{2}}{2}\right),j=1,2,$$

where $\hat{u}=u/∥u∥$ and

(50) $$u=\left(\begin{array}{c} 1\\ \left(\frac{\hbar \Gamma^{1/2}}{mc}\right)\frac{K_{ji}}{1+\gamma } \end{array}\right)$$

is the two component spinor for a spin up electron. This is then used in the symmetrized wavefunction for the two bosons and electrons, respectively.

## 3. Flight Times of Identical Non-Relativistic Particles Scattered by a Delta Function Barrier

### 3.1. Preliminaries

The Hamiltonian for the delta function barrier is:

(51) $$\hat{H}=-\frac{\hbar^{2}}{2M}\frac{d^{2}}{dx^{2}}+\varepsilon \delta \left(x\right)$$

and the coupling coefficient ε>0. The eigenfunctions of the Hamiltonian at energy

(52) $$E=\frac{\hbar^{2}k^{2}}{2M}$$

are

(53) $$\psi \left(x\right)=\left(\begin{array}{l} \exp\left(ikx\right)+R\left(k\right)\exp\left(-ikx\right),\;\;\;x<0\\ \;T\left(k\right)\exp\left(ikx\right),\;\;\;\;\;\;\;\;\;\;\;x>0, \end{array}\right)$$

with the reflection amplitude given as:

(54) $$R\left(k\right)=\frac{-i\alpha \left(k\right)}{1+i\alpha \left(k\right)},\;\;\;\alpha \left(k\right)=\frac{M\varepsilon }{\hbar^{2}k}.$$

The transmission amplitude is:

(55) $$T\left(k\right)=\frac{1}{1+i\alpha \left(k\right)}$$

and one readily sees that

(56) $${\left|R\left(k\right)\right|}^{2}+{\left|T\left(k\right)\right|}^{2}=1.$$

The phase time delays are defined to be:

(57) δtT,R=MℏkIm1Y∂Y∂k,Y=R,T.

Using the reduced variables as in Equation (4) and the reduced delta function coupling variable,

(58) $$\epsilon =\frac{M\varepsilon }{\hbar^{2}\sqrt{\Gamma }},$$

the transmission and reflection probabilities become:

(59) $${\left|T\left(K\right)\right|}^{2}=\frac{K^{2}}{K^{2}+\epsilon^{2}},{\left|R\left(K\right)\right|}^{2}=\frac{\epsilon^{2}}{K^{2}+\epsilon^{2}}$$

and the phase times are:

(60) $$\delta \tau_{T}=\delta \tau_{R}=\frac{\epsilon }{K\left[K^{2}+\epsilon^{2}\right]}\equiv \delta \tau .$$

The phase time then implies that for a repulsive delta function potential (ϵ>0) the flight time is lengthened while for an attractive delta function potential it is shortened. In the limit that the coupling coefficient ϵ→∞, which is the equivalent of a hard wall potential, the transmission amplitude vanishes while the reflection amplitude goes to −1. The reflection time delay vanishes in this case, the interference of the forward and reflected wave does not change the reflected phase time delay. For a fixed nonzero value of ϵ>0 in the limit that the energy vanishes (K→0) the delay diverges as $K^{-1}$. For an attractive delta function potential, the flight time is shortened and the reduction diverges as $K^{-1}$. Due to the zero width of the delta function potential, the dwell time [27] in the barrier always vanishes.

It is worthwhile here also to consider the imaginary time defined as [28]:

(61) tim,R,T=ℏRe1Y∂Y∂E,Y=R,T

so that the (reduced) transmitted imaginary time is positive

(62) $$\tau_{im,T}=\frac{\epsilon^{2}}{K^{2}\left(K^{2}+\epsilon^{2}\right)}$$

while the reflected imaginary time is negative

(63) $$\tau_{im,R}=-\frac{1}{\left(K^{2}+\epsilon^{2}\right)}.$$

As the momentum increases, the transmission probability increases while the reflection probability decreases, so that the transmitted imaginary time is positive, and the reflected is negative.

#### 3.1.1. The Single Particle Dynamics. Momentum Filtering

Initially we consider a Gaussian wavepacket as in Equation (3) whose momentum representation is:

(64) $$\Psi \left(k,0\right)={\left(\frac{1}{\pi \Gamma }\right)}^{1/4}\exp\left(-\frac{{\left(k-k_{i}\right)}^{2}}{2\Gamma }-ikx_{i}\right).$$

The time dependent wavepacket in the transmitted region is:

(65) $$\Psi_{T}\left(x,t\right)=\int_{-\infty }^{\infty }\frac{dk}{\sqrt{2\pi }}\Psi \left(k,0\right)T\left(k\right)\exp\left(ikx-i\frac{\hbar k^{2}}{2M}t\right),x\geq 0$$

and in the reflected region is:

(66) $$\Psi_{R}\left(x,t\right)=\int_{-\infty }^{\infty }\frac{dk}{\sqrt{2\pi }}\exp\left(-i\frac{\hbar k^{2}}{2M}t\right)\Psi \left(k,0\right)\left[\exp\left(ikx\right)+R\left(k\right)\exp\left(-ikx\right)\right],x\leq 0.$$

Using the reduced variables as in Equations (4) and (58) and carrying out the momentum integrations in Equations (65) and (66) one finds that the transmitted time-dependent wavepacket (X≥0) is:

(67) $$\begin{array}{ccc} & & \Psi_{T}\left(X,\tau \right)=\Psi_{fp}\left(X,\tau \right)\\ & & \left[1-\epsilon \frac{\sqrt{\pi \left(1+i\tau \right)}}{\sqrt{2}}\exp\left[-\frac{\left(1+i\tau \right)}{2}{\left(Z_{T}+i\epsilon \right)}^{2}\right]\mathrm{erf}c\left(-\frac{i\sqrt{\left(1+i\tau \right)}}{\sqrt{2}}\left(Z_{T}+i\epsilon \right)\right)\right], \end{array}$$

where $\Psi_{fp}\left(X,\tau \right)$ is the free particle time-dependent wavepacket as in Equation (16), erfc is the complementary error function, and

(68) $$Z_{T}=\frac{\left[K_{i}+i\left(X-X_{i}\right)\right]}{\left(1+i\tau \right)}.$$

The reflected time-dependent wavefunction (X≤0) is

(69) $$\begin{array}{ccc} & & \Psi_{R}\left(X,\tau \right)=\Psi_{fp}\left(X,\tau \right)\\ & & -\Psi_{fp}\left(-X,\tau \right)\epsilon \frac{\sqrt{\pi }}{\sqrt{2}}\sqrt{\left(1+i\tau \right)}\exp\left(-\frac{\left(1+i\tau \right)}{2}{\left(i\epsilon +Z_{R}\right)}^{2}\right)\mathrm{erfc}\left(-i\sqrt{\frac{\left(1+i\tau \right)}{2}}\left(i\epsilon +Z_{R}\right)\right), \end{array}$$

with

(70) $$Z_{R}=\frac{\left[K_{i}-i\left(X+X_{i}\right)\right]}{\left(1+i\tau \right)}.$$

In practice, if the incident (reduced) momentum is sufficiently large, which will be the case in all of our computations, and since we will be using small momentum variances, one may safely replace the complementary error function with its asymptotic expansion so that to leading order

(71) $$\Psi_{T}\left(X,\tau \right)≃\Psi_{fp}\left(X,\tau \right)\left[\frac{Z_{T}}{\left(Z_{T}+i\epsilon \right)}\right],X\geq 0$$

and

(72) $$\Psi_{R}\left(X,\tau \right)≃\Psi_{fp}\left(X,\tau \right)-\Psi_{fp}\left(-X,\tau \right)\frac{\epsilon }{\left(\epsilon -iZ_{R}\right)},X\leq 0.$$

Equations (71) and (72) will be the ‘workhorses’ for the numerical implementations below, but we stress that we have checked the validity of the asymptotic expansion and it is quantitative for the conditions used here. To see the long time limit we note that

(73) $${\left|\frac{Z_{T}}{\left(Z_{T}+i\epsilon \right)}\right|}^{2}=\frac{\left[K_{i}^{2}+{\left(X-X_{i}\right)}^{2}\right]}{\left[{\left(K_{i}-\epsilon \tau \right)}^{2}+{\left(X-X_{i}+\epsilon \right)}^{2}\right]}$$

and this goes as $\tau^{-2}$ in the long time limit so that the transmitted single particle density decays as $\tau^{-3}$. The calculation is a bit more involved for the reflected density but it also decays as $\tau^{-3}$. In contrast to the free particle, due to the potential, the mean flight time for the density and the flux (Equations (15 ) is well-defined (even when the time integral in the denominator of Equation (13 has the limits [0,∞]).

The time ($\tau_{fp}$) it would take a free particle initiated at $X_{i}$ with momentum $K_{i}$ to reach the screen located at X>0 is

(74) $$\tau_{fp}=\frac{X-X_{i}}{K_{i}}.$$

With this notation and $\epsilon_{i}\equiv \epsilon /K_{i}$, we may rewrite the single particle density in the transmitted region as:

(75) $${\left|\Psi_{T}\left(X,\tau \right)\right|}^{2}≃\frac{\left(1+\tau_{fp}^{2}\right)}{\sqrt{\pi \left(1+\tau^{2}\right)}}\exp\left(-\frac{K_{i}^{2}{\left[\tau_{fp}-\tau \right]}^{2}}{\left(1+\tau^{2}\right)}-\ln\left[{\left(\epsilon_{i}\tau -1\right)}^{2}+{\left(\tau_{fp}+\epsilon_{i}\right)}^{2}\right]\right)$$

and ask when the exponent is maximized as a function of the reduced time τ. Defining

(76) $$G\left(\tau \right)=\frac{K_{i}^{2}{\left[\tau_{fp}-\tau \right]}^{2}}{\left(1+\tau^{2}\right)}+\ln\left[{\left(\epsilon_{i}\tau -1\right)}^{2}+{\left(\tau_{fp}+\epsilon_{i}\right)}^{2}\right],$$

we note that

(77) $$\frac{dG\left(\tau \right)}{d\tau }=-2\frac{K_{i}^{2}\left[\tau_{fp}-\tau \right]}{\left(1+\tau^{2}\right)}-2\tau \frac{K_{i}^{2}{\left[\tau_{fp}-\tau \right]}^{2}}{{\left(1+\tau^{2}\right)}^{2}}+\frac{\epsilon_{i}2\left(\epsilon_{i}\tau -1\right)}{\left[{\left(\epsilon_{i}\tau -1\right)}^{2}+{\left(\tau_{fp}+\epsilon_{i}\right)}^{2}\right]}.$$

Setting the derivative equal to zero,

(78) $$\frac{K_{i}^{2}\left[\tau_{fp}-\tau \right]}{\left(1+\tau^{2}\right)}+\tau \frac{K_{i}^{2}{\left[\tau_{fp}-\tau \right]}^{2}}{{\left(1+\tau^{2}\right)}^{2}}=\frac{\epsilon \left(\epsilon \tau -K_{i}\right)}{\left[{\left(\epsilon \tau -K_{i}\right)}^{2}+{\left(K_{i}\tau_{fp}+\epsilon \right)}^{2}\right]},$$

looking for a solution

(79) $$\tau =\tau_{fp}\left(1-\Delta \tau \right),$$

remembering that $\tau_{fp}\gg 1$ and assuming that Δτ≪1 leads to the solution (see Equation (62))

(80) $$\Delta \tau ≃\frac{\epsilon^{2}}{K_{i}^{2}\left[K_{i}^{2}+\epsilon^{2}\right]}=\tau_{im,T}\left(K_{i}\right),$$

and this is precisely the momentum filtering effect. Due to the increase of the transmission probability with energy, the high-energy components of the incident wavepacket are preferably transmitted so that the flight time is reduced [29].

#### 3.1.2. Two Particles Dynamics

The composite initial wavefunction of the two particles is given by Equation (6) For the δ-tunneling dynamics, the time evolved wavefunction will have four components, corresponding to the two particles being in the reflected region or the transmitted region or that one particle is in the reflected region and the other in the transmitted region and vice versa:

(81) $$\begin{array}{ccc} & & \Psi_{\alpha }\left(X_{1},X_{2},\tau \right)\\ & = & \left[\Psi_{1,T}\left(X_{1},\tau \right)\Psi_{2,T}\left(X_{2},\tau \right)+\cos\left(\pi \alpha \right)\Psi_{2,T}\left(X_{1},\tau \right)\Psi_{1,T}\left(X_{2},\tau \right)\right]\theta \left(X_{1}\right)\theta \left(X_{2}\right)\\ & & +\left[\Psi_{1,R}\left(X_{1},\tau \right)\Psi_{2,R}\left(X_{2},\tau \right)+\cos\left(\pi \alpha \right)\Psi_{2,R}\left(X_{1},\tau \right)\Psi_{1,R}\left(X_{2},\tau \right)\right]\theta \left(-X_{1}\right)\theta \left(-X_{2}\right)\\ & & +\left[\Psi_{1,T}\left(X_{1},\tau \right)\Psi_{2,R}\left(X_{2},\tau \right)+\cos\left(\pi \alpha \right)\Psi_{2,T}\left(X_{1},\tau \right)\Psi_{1,R}\left(X_{2},\tau \right)\right]\theta \left(X_{1}\right)\theta \left(-X_{2}\right)\\ & & +\left[\Psi_{1,R}\left(X_{1},\tau \right)\Psi_{2,T}\left(X_{2},\tau \right)+\cos\left(\pi \alpha \right)\Psi_{2,R}\left(X_{1},\tau \right)\Psi_{1,T}\left(X_{2},\tau \right)\right]\theta \left(-X_{1}\right)\theta \left(X_{2}\right), \end{array}$$

where $\Psi_{1,T}$, $\Psi_{2,T}$, $\Psi_{1,R}$ and $\Psi_{2,R}$ are the transmitted and reflected wave functions for each particle when considered to be independent as given in Equations (67) and (69). The density and flux of particles reaching the screen located at *X* are then given by Equations (11) and (12), respectively. The respective density and flux mean flight times are obtained, using Equation (15). As mentioned above, these distributions and mean times are well defined under the presence of a potential since the density decays at long times as $\tau^{-3}$. From this expression, one-particle time distributions are obtained by integrating over the second particle and vice versa. Due to the fact the physics is the same, the resulting time distributions should be multiplied by a factor two.

## 4. Numerical Results

To analyze the role played by the symmetry of the total wave function in systems of identical particles when considering free dynamics and tunneling from a delta barrier, we have identified two sets of initial conditions (in reduced coordinates): (I) different locations with the same momenta, $X_{1i}=-301$, $X_{2i}=-299$ with $K_{1i}=K_{2i}=10$ and (II) the same locations with different momenta, $X_{1i}=X_{2i}=-300$ with $K_{1i}=10.1$ and $K_{2i}=9.9$. When the differences in initial positions and momenta differ more, the cross terms in the density of the two-particle system become smaller, and one rapidly reaches the distinguishable particle limit. Unless otherwise stated, we will assume Γ=0.01 for the initial width of the Gaussian functions and ε=1, so that the reduced value of the coupling parameter to the delta barrier (Equation (58) will be ϵ=10 in both cases. In all cases, the position of the screen is at $X_{f}=\pm 450$ and the delta barrier is located at the origin.

### 4.1. Free Particle Non-Relativistic Flight Times

**Figure 1:**
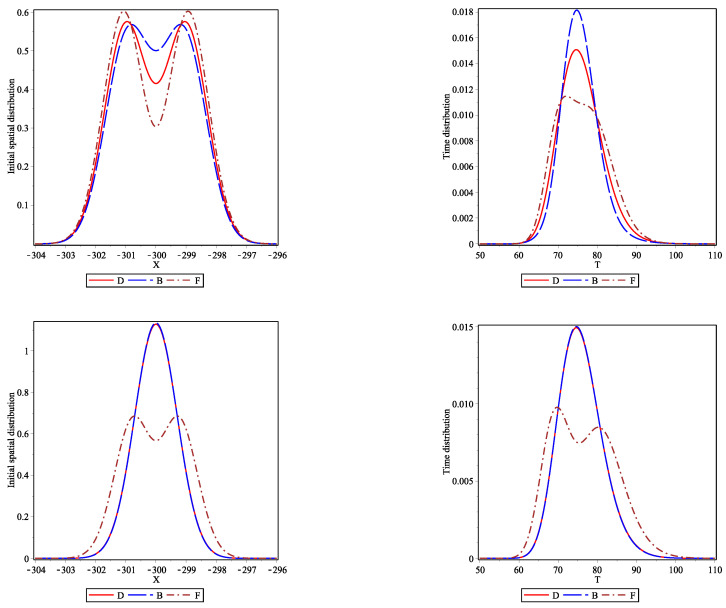
Non-relativistic free particle dynamics. The left panels show the initial spatial density distribution. The right panels show the resulting one particle flight time distributions as defined in Equation (11) hitting the screen located at $X_{f}=450$. The other parameters used are Γ=0.01 for the initial width of the coherent states; for the set (I) (initial spatial difference) $X_{1i}=-301$, $X_{2i}=-299$, $K_{1i}=K_{2i}=10$, left and right top panels; and for the set (II) (initial momentum difference) $X_{1i}=X_{2i}=-300$, $K_{1i}=10.1$, $K_{2i}=9.9$, left and right bottom panels.

In Figure 1, we plot the initial spatial distributions (left panels) and the one-particle flight time distributions (right panels) for the two sets of initial conditions (see Equation (11). The top two panels are for the set of initial conditions (I) (initial spatial difference) and the bottom two are for the set (II) (initial momentum difference). The solid red curve is used for distinguishable particles, the long-dashed blue curve for bosons and the dot-dashed brown curve for fermions. The one-particle flight time distribution is always broader for fermions displaying early and late arrivals. Bosons tend to arrive later than distinguishable particles, showing the narrowest time distribution. Furthermore, the flight time distributions of distinguishable particles and bosons tend to have a similar shape, losing the two lobes of the initial density, whereas the fermions display a double-peaked time distribution, reflecting their initial density. In the bottom-right panel, bosons and distinguishable particles behave essentially identically whereas fermions display a bimodal time distribution. Fermions not only arrive at the screen earlier and later, there is a distinctive time asymmetry in the flight time distribution. We attribute these different behaviors to the bunching and anti-bunching properties of bosons and fermions, respectively.

**Figure 2:**
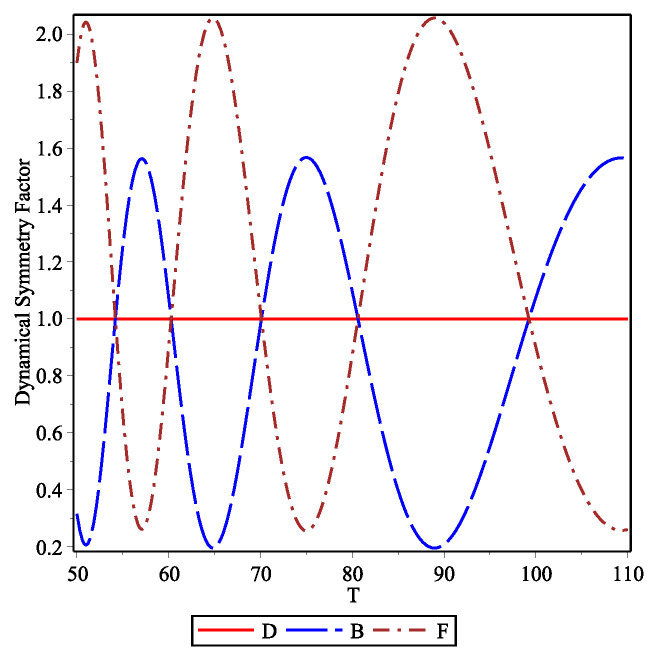
Dynamical symmetry factor versus time at $X_{f}=450$ for the set of initial conditions (I). Red line is for distinguishable particles, long-dashed blue curve for bosons and dotted-dashed brown curve for fermions.

These global behaviors can be better understood from Figure 2 where the DS factor is plotted for bosons and fermions for the set of initial conditions (I). The red line is for distinguishable particles where no symmetry is displayed, the long-dashed blue and dotted-dashed brown curves are for bosons and fermions, respectively. In the time window where particles are arriving at $X_{f}=450$, we observe that this factor is an oscillatory function of time which is just in opposite phase for the two indistinguishable particles. Furthermore, in the time window of interest, fermions display two oscillations whereas bosons only one oscillation which is placed in between the fermionic oscillations. As mentioned above, this DS factor reflects the dynamical bunching and anti-bunching effects. The early and late arrival of fermions at the screen is not related only to the ‘front’ of the initial fermionic density distribution; it also results from the different dynamical evolution of the indistinguishable particles.

**Figure 3:**
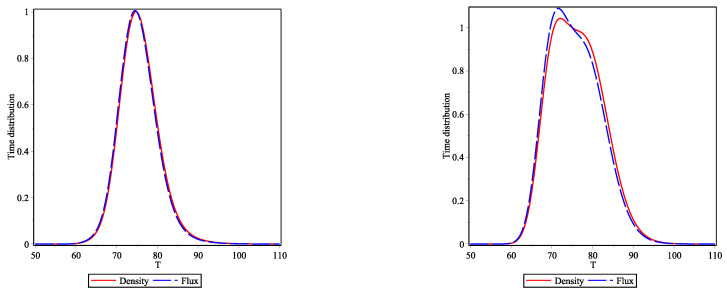
One-particle density (solid red curves) and flux (blue long-dashed curves) flight time distributions at $X_{f}=450$ for bosons (**left panel**) and fermions (**right panel**).

It is well known that, in the literature on arrival times, the information coming from the fluxes is used instead of the densities. Here, we would like only to stress that this information is nearly identical in both cases for the initial conditions we have used. In Figure 3, we plot the one-particle flight time distribution obtained from the density (solid red curves) and flux (blue long-dashed curves) distributions at $X_{f}=450$ for bosons (left panel) and fermions (right panel). For bosons, both distributions are essentially identical except for the negligible long time tails whereas for fermions there is a small difference in the region of the maximum of the time distribution. The bimodal character is kept for both curves, albeit for the density it is slightly more pronounced.

**Figure 4:**
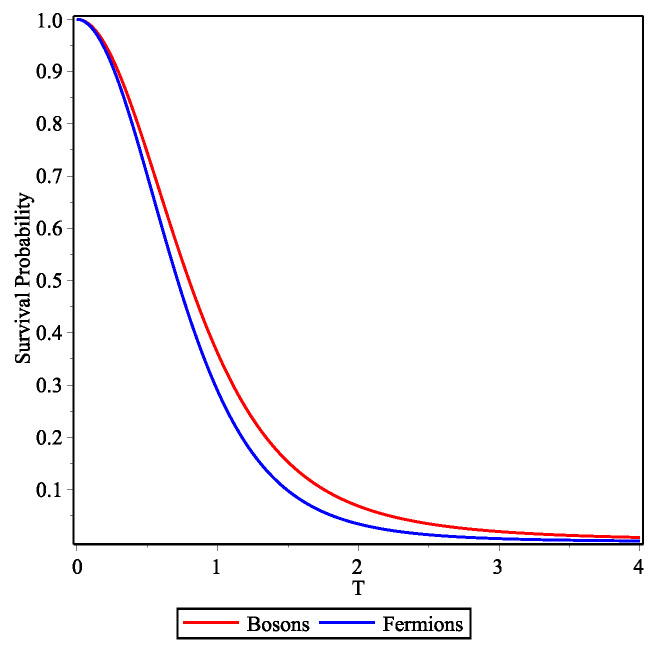
Survival probability of bosons (red curve) and fermions (blue curve) for non-relativistic free particles with $K_{i}=1$. The initial width of the Gaussians here is taken to be Γ=0.01.

Another interesting aspect is the analysis of the survival probability given by Equations (42)–(44) and the limits at small initial spatial $\Delta_{X}$ and momentum $\Delta_{K}$ differences. When the mean incident momentum is large (as in the two cases considered above), the survival probability decays very rapidly and the differences between bosons and fermions is negligible; or, putting it differently they show up at times at which the survival probability is already very small. However, if one chooses say $K_{i}=1$ then the differences are noticeable, as seen in Figure 4. The decay of the bosonic survival probability is slower than that of the fermionic.

### 4.2. Free Particle Relativistic Flight Time Distributions

**Figure 5:**
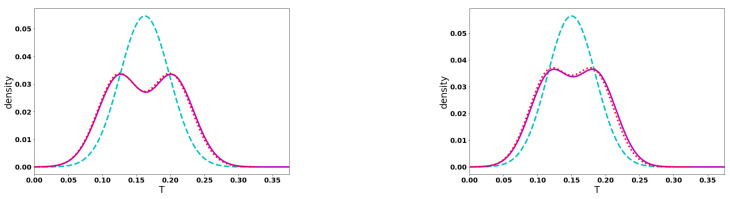
Time-dependent flight time density distributions for two photons (dashed lines) and two relativistic electrons (solid lines). The left and right panels are for initial spatial (case I) and momentum (case II) differences, respectively. The initial width of the Gaussians here is taken to be Γ=0.0025; also shown (dotted lines) are the densities that would be seen if the electrons traveled dispersion-free at the speed of light.

Figure 5 shows the time-dependent density at the screen (at X=0) for two photons and two electrons traveling near the speed of light. In the left panel, the two Gaussians are centered at $X_{1i}=-3.5$ and $X_{2i}=-3$ and at a wavenumber consistent with velocity, v=0.99c. In the right panel, the two Gaussians are centered at $X_{1i}=X_{2i}=-3$ and at wavenumbers consistent with v=0.984c and 0.996c. In both cases, an electron is more likely to arrive at the screen before a photon. However, this is due to the fact that the initial density for the electrons is broader than that of the photons. To show this, the density that would be seen if the electrons traveled dispersion-free at the speed of light is also shown (dotted lines). The observed electron density clearly travels with a speed less than *c*. The early and late arrivals of the fermions are just a reflection of their initial density, which, as may be seen from the left panels of Figure 1, is broader than the initial distribution for bosons, due to the anti-bunching effect of fermions. More subtle dynamical contributions to early and late arrival likelihood are suppressed in the relativistic regime, since dispersion and associated wavepacket broadening are much reduced.

### 4.3. Identical Particle, Non-Relativistic Flight Times for Scattering on a Delta Potential Barrier

In this case, as noted above, the mean flight times (Equation (15) for the density are well defined, and they provide a clear measure of the effect of particle symmetry on the flight time. In Table 1, we provide the transmitted and reflected mean flight times (in reduced coordinates) for bosons $<\tau_{1,B}{>}_{T,R}$ and fermions $<\tau_{1,F}{>}_{T,R}$, with Γ=0.01 and ϵ=10 for the two sets of initial conditions, (I) and (II). Arrival times at the delta barrier are around 30 and at the screen around 75 (in reduced units) for the set of initial conditions (I). Transmitted mean times are always shorter than reflected mean times due to the momentum filtering effect and those of fermions are always greater than for bosons due to their respective anti-bunching and bunching properties.

**Figure 6:**
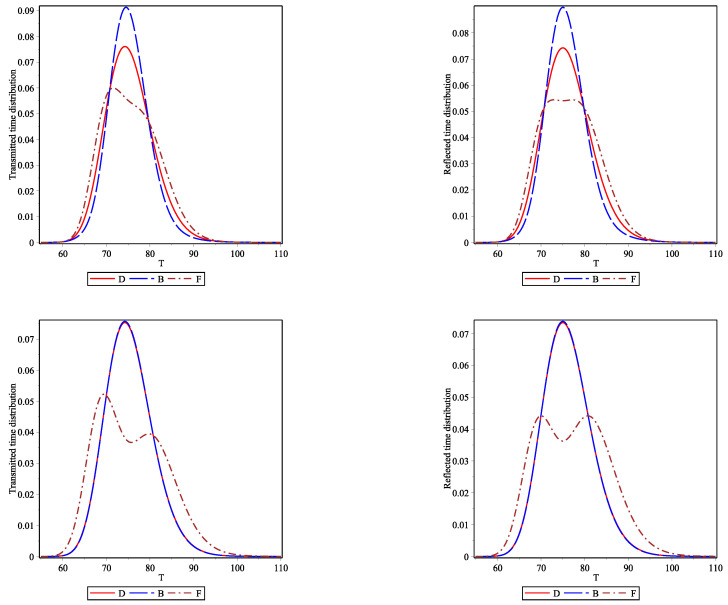
Tunneling δ-barrier dynamics with Γ=0.01. One-particle transmitted and reflected density based flight time distributions for conditions (I) are shown in the left and right top panels and for conditions (II) in the left and right bottom panels.

The corresponding flight time probability distributions (Equation (13) are shown in Figure 6. The top and bottom panels correspond to initial spatial (case I) and initial momentum (case II) differences, the left and right panels correspond to the transmitted and reflected flight time distributions, respectively. The trends are similar to those found in the free particle dynamics scenario. The effect of symmetry on flight times seems to be robust and is not changed much in the presence of an interaction barrier. Time distributions are broadest for fermions and narrowest for bosons, with distinguishable particles in between. The asymmetry of the bimodal reflected distributions for fermions is not as strong as for the transmitted distributions.

In previous work, where we studied the MacColl–Hartman effect, we argued against the ‘front’ of the wavepacket being used to explain away supposedly superluminal propagation for *tunneling* particles [19,24], noting that the superluminality cannot be used for the purpose of early signaling. When considering the MacColl-Hartman effect, one is comparing final time distributions of tunneled particles with free particles, but the two have the same initial density distribution. In the cases considered here, the initial wavepacket of the fermions is broadened when compared to that of the bosons. It is this broadening which leads to early and late arrival times of fermions as compared to bosons, so that one does not have to consider here the possibility of superluminality.

**Figure 7:**
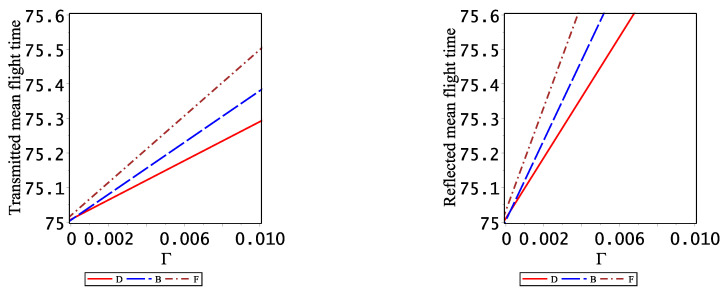
One-particle transmitted (**left panel**) and reflected (**right panel**) mean flight times versus Γ for distinguishable particles, bosons and fermions under conditions (I). The legend is the same one used along this work for each particle.

Finally, it is also of interest to analyze the influence of the initial width (Γ) of the Gaussian wavepackets on the mean tunneling flight times which are well defined, especially when Γ approaches zero such that the spatial extent of the two Gaussians is large, creating large initial overlaps. This analysis is carried out by means of a fitting procedure to a linear function (bΓ+c) obtained from the numerics for eight values of the initial width, $\Gamma =10^{-2},0.25\;\times 10^{-2},9.0\;\times 10^{-4},4.0\;\times 10^{-4},10^{-4},0.25\;\times 10^{-4},9.0\;\times 10^{-6},4.0\;\times 10^{-6}$. The reduced distances (*X*), momenta (*K*) and times (τ) are scaled as in Equations (4) and the coupling constant (ϵ) as in Equation (58) relative to their values with Γ=0.01. In Figure 7, the transmitted (left panel) and reflected (right panel) mean (density based) flight times are plotted as functions of the spatial width parameter Γ for distinguishable particles (solid red line), bosons (dashed blue line) and fermions dashed-dotted brown line). In all cases, the quality of the linear fit is very good. All mean flight times tend to a value of $\langle \tau \rangle =75.005\pm 10^{-6}$. Under the conditions for cases I and II the free particle time is 75, the extra amount of 0.005 reflects the phase time (Equation (60)), which is 0.005. Thus, the particle symmetry does not affect the mean tunneling flight time. In this limit of large spatial extent (Γ→0) of the initial Gaussian wave functions, the mean flight time tends to the phase time for distinguishable particles.

## 5. Discussion

In this work, we studied the influence of the symmetry of the wavefunction for a system consisting of two non-interacting identical particles on the flight time in free evolution and when tunneling by a δ-barrier. In free evolution, whether considering density or flux based flight times, the well-known bunching and anti-bunching properties exhibited by bosons and fermions respectively have been quantified in terms of a DS factor which depends on the initial conditions, distances and time. This factor reflects the different dynamical effects found for bosons and fermions. Furthermore, by analyzing the short-time dynamics through the survival probability, we find that fermions tend to decay faster than bosons, and this should have profound implications for the well-known Zeno effect. For the scattering by a δ-barrier, the symmetry of the wave function also affects the flight time distributions and mean tunneling flight times. For a finite initial spatial width of the wavepacket the mean flight times are greater for bosons and fermions than for distinguishable particles. However, in the limit of an infinitely narrow initial momentum distribution (Γ→0) the symmetry seems to play no role.

In our model, we have considered non-interacting identical particles. The importance of the symmetry of the system has not been addressed for flight times and the Zeno effect even though it should be readily accessible. This is clearly an over-simplification of the real problem since, for example, electrons interact with each other through the long-range Coulomb repulsion. For example, the triplet states of two electrons will give a fermionic spatial wavefunction while the singlet state a bosonic one. In a scattering experiment similar to the one discussed in Refs. [8,9], the two particles in the center of mass frame will approach each other such that the distance between the two particles becomes small and the symmetry will affect the time of flight distribution of the two particles. Due to the anti-bunching property of fermions, one should expect broader temporal distributions of the scattered particles.

Lozovik et al. [30] considered tunneling of two interacting particles in a double-well potential. They used quantum molecular dynamics within the Wigner representation and found that exchange effects are very important and affect the tunneling. However, this work does not mention flight time distributions. It is thus possible, at least in principle, to study the effect of particle symmetry on flight time distributions of identical electrons, without neglecting the repulsive potential of interaction between them, though the actual numerical implementation, especially in the relativistic regime is much more challenging.

Finally, we note that the study of symmetry on flight time distributions presented in this paper may be generalized to anyons, by introducing the appropriate phase in the initial distribution.

## Figures and Tables

**Table 1:** Initial conditions and one-particle transmitted (T) and reflected (R) mean flight times (in reduced coordinates) for bosons, $<\tau_{1,B}{>}_{T,R}$, and fermions, $<\tau_{1,F}{>}_{T,R}$, scattered by a δ- barrier with Γ=0.01 and ϵ=10.

$X_{1i}$	$X_{2i}$	$X_{f}$	$K_{1i},K_{2i}$	$<\tau_{1,B}{>}_{T}$	$<\tau_{1,B}{>}_{R}$	$<\tau_{1,F}{>}_{T}$	$<\tau_{1,F}{>}_{R}$
−301	−299	± 450	10, 10	75.2908	75.8847	75.4992	76.5237
−300	−300	± 450	10.1, 9.9	75.3749	76.1740	76.7417	77.3525

## Data Availability

Not applicable.
